# The association between dietary acid load and odds and severity of irritable bowel syndrome in adults

**DOI:** 10.1038/s41598-022-23098-9

**Published:** 2022-11-08

**Authors:** Fatemeh Mobasheri, Farzad Shidfar, Azadeh Aminianfar, Ammar Hassanzadeh Keshteli, Ahmad Esmaillzadeh, Peyman Adibi

**Affiliations:** 1 Department of Nutrition, School of Public Health, Iran University of Medical Sciences, Tehran, Iran; 2 Research Center for Biochemistry and Nutrition in Metabolic Diseases, Kashan University of Medical Sciences, Kashan, Iran; 3 Department of Medicine, University of Alberta, Edmonton, Canada; 4 Department of Community Nutrition, School of Nutritional Sciences and Dietetics, Tehran University of Medical Sciences, Tehran, Iran; 5 Obesity and Eating Habits Research Center, Endocrinology and Metabolism Molecular-Cellular Sciences Institute, Tehran University of Medical Sciences, Tehran, Iran; 6 Department of Community Nutrition, School of Nutrition and Food Science, Isfahan University of Medical Sciences, Isfahan, Iran; 7 Integrative Functional Gastroenterology Research Center, Isfahan University of Medical Sciences, Isfahan, Iran

**Keywords:** Diseases, Gastroenterology

## Abstract

No study has been conducted to investigate the association between dietary acid load and irritable bowel syndrome (IBS). So, this cross-sectional study was performed to investigate the association between dietary acid load and odds of IBS, its severity, and IBS subtypes. A sample of 3362 Iranian subjects was selected from health centers in Isfahan province. A validated semi-quantitative food frequency questionnaire (DS-FFQ) was applied to estimate dietary intakes. The dietary acid load was measured using net endogenous acid production (NEAP), dietary acid load (DAL), and potential renal acid load (PRAL) scores. In crude models, the highest compared with the lowest category of the PRAL score was significantly associated with increased odds of IBS severity in participants with BMI ≥ 25 (kg/m^2^) (OR = 1.54; 95% CI = (1.03–2.32). Also, the results indicated a significant positive association between the PARL and odds of mixed subtype of IBS (OR = 1.74; 95% CI = (1.11–2.74); *P* trend = 0.02). In propensity score-adjusted model with potential confounders, only a positive association was found between PRAL and odds of mixed subtype of IBS (OR = 1.78; 95% CI = (1.05–3.00); *P* trend = 0.03). The DAL and NEAP scores tended to show non-significant similar findings. This study indicates that dietary acid load might be associated with odds of mixed type of IBS. However, further research is warranted to infer these findings.

## Introduction

Irritable bowel syndrome (IBS) is a widespread bowel disorder identified by changes in bowel habits along with abdominal pain or discomfort^1,2^. This untreated pain can lead to poor quality of life, daily functional disability, loss of work, and social stresses^3^. The global prevalence of this disorder is 9.2% and 3.8% based on the Rome III and Rome IV criteria, respectively ^4^. Factors including stress, inflammation, abnormal gut flora, and gut sensorimotor dysfunction may be relevant to the pathogenesis of the IBS^5^. Also, it has been suggested that other factors such as dietary items, extensive laxative usage, antibiotics, caffeine, previous gastrointestinal (GI) disease, and irregular sleep rhythm may severe the complications of the disease^6^. Among these, dietary factors have been proposed to have undeniable effects on the occurrence and severity of symptoms in patients with IBS^7^.

One of the important dietary components that might be linked with IBS complications is the use of acidogenic dietary regimens. Accordingly, it seems a low fermentable oligosaccharide disaccharide monosaccharide and polyol (FODMAP) diet, as a known dietary approach in the treatment of IBS disease, may alleviate the symptoms of patients with IBS by reducing luminal acidity. The fermentation metabolites generated by FODMAP metabolism are usually acidic compounds (acetate, hydrogen sulfide, lactate, methane, hydrogen, and amines), which may contribute to increased luminal acidity^8^. Animal experiments showed that intraluminal colonic acidic hypertonic saline infusions (which occur in carbohydrate malabsorption) increased visceral hypersensitivity^9^. The inconsistent research also found increased gut immune activation, a possible mechanism in the pathogenesis of the IBS disease, following FODMAPs administration^10,11^. In addition, an acidogenic and alkaline-deficient Western dietary pattern has been linked to the endogenous acid generation and metabolic acidosis^12^. The metabolic acidosis can damage tissues which subsequently triggers inflammation^13–15^. For practical purposes three scores were established to estimate dietary acid load; the potential renal acid load (PRAL) score (which includes five nutrients such as potassium, phosphorus, magnesium, protein, and calcium), dietary acid load (DAL), and the net endogenous acid production (NEAP) (takes into account two nutrients, protein, and potassium)^16–18^. Interestingly, previous studies showed positive associations between the dietary acid load and increased risk of hypertension^19^, insulin resistance^20^, type 2 diabetes^21^, and non-alcoholic fatty liver^22^. However, as far as we know no study has examined the relationship between dietary acid load and IBS risk; the studies showed positive associations between this index and inflammation^23^ and stress^24^, as major risk factors in the pathogenesis of this disease.

In general, there is limited evidence indicating the potential effects of the acidity of a diet on the odds and severity of complications in patients with IBS. Also, some studies showed greater acidity in a diet following increased consumption of refined grains and salt^25^. Unfortunately, the intake of these food items is high in Iran^26^ which can lead to higher dietary acid load and subsequent complications-related disease. Thus, this study aimed to investigate the associations between the dietary acid load and odds and severity of IBS in a sample of Iranian adults participating in the Study on the Epidemiology of Psychological, Alimentary Health, and Nutrition (SEPAHAN) project.

## Materials and methods

### Participants

An analysis of this cross-sectional study was based on the SEPAHAN project (Epidemiology of Psychological, Alimentary Health, and Nutrition), a study that aimed to examine lifestyle and psychological factors with functional GI disorders among a sample of adults in Isfahan province, Iran. Definite data about the SEPAHAN project was published previously^27^. This project was conducted among non-scholarly staff (matured 18–55 years) who were working in 50 diverse medical care places subsidiary with Isfahan University of Medical Sciences (IUMS). The included population were service staff, employees, and managers and their socioeconomic status was similar to the general Iranian population. The whole project was performed in two separate phases to enhance the accuracy of data collection. Self-administered questionnaires were provided to 10,087 subjects in the first phase to gather data on demographics, medical history, food intake, physical activity, and anthropometric measures. The questionnaires were returned by 8691 individuals at the end of the first phase (response rate: 86.16 percent). Participants were asked to provide data on their GI health in the second phase of the study (response rate: 64.6 percent). When the data from both periods were combined, 4633 participants were found with comprehensive information on dietary intakes and GI diseases. Finally, Participants with daily energy intakes outside of the range of 800–4200 kcal and missing data on any relevant variables were eliminated. Accordingly, we analyzed information about 3,362 participants. This study was performed in line with the principles of the Declaration of Helsinki. All the participants didn't receive any diet, especially the FODMAP diet. All participants were asked to complete a written informed consent form before the initiation of the study. The study protocol was ethically approved by the Bioethics Committee of IUMS, Isfahan, Iran (Approval No. 189069, 189082, and 189086).

### Dietary intakes assessment and DAL scores calculation

To gather dietary data, a semi-quantitative Willett-format dish-based food frequency questionnaire (DS-FFQ) was employed. This validated DS-FFQ included 106 food items intended for use by Iranian adults^28^. It consists of a five-category list of foods and dishes: "mixed dishes" are comprised of 29 distinct cooked and canned dishes; "grains" include different kinds of bread, cakes, biscuits, and potatoes (10 items); “dairy products” include dairies, butter, and cream (9 items); “fruits and vegetables” (22 items); “miscellaneous food items and beverages” are comprised of sweets, fast foods, nuts, desserts, and beverages (36 items). The most often eaten portion size for these 106 food items and combination meals was determined, and participants reported their dietary intakes using nine multiple-choice frequency response categories ranging from "never or 1/month" to "12/day". These frequency response categories varied from 6 to 9 choices. The high-frequency category was omitted for foods consumed infrequently while the number of multiple-choice categories increased for common foods with high consumption. Finally, food items were converted into grams by computing the number of portion sizes based on household measurements and taking into account how often each dietary item is consumed. Potential acidity of the dietary intakes were calculated using the PRAL (PRAL (mEq/d) = (protein [g/d] × 0.49) + (phosphorous [mg/d] × 0.037)−(potassium [mg/d] × 0.021)−(calcium [mg/d] × 0.013)−(magnesium [mg/d] × 0.026))^16,29^, the NEAP (NEAP (mEq/d) = (54.5 × protein intake [g/d]/potassium intake [mEq/d])-10.2) scores^17^, and the DAL(mEq/day) = PRAL + (body surface area [m2] × 41 [mEq/day]/1.73 m^2^)^16–18^. We calculated body surface area using the Du Bois formula: 0.007184 × height 0.725 × weight 0.425^30,31^.

### Assessment of IBS

To assess IBS symptoms in the study population, we used a modified Persian version of the Rome III questionnaire^32^. Because most participants were unable to differentiate between the descriptors used in the original questionnaire (never, once a month, twice a month, three times a month, once a week, more than once a week, and every day), a 4-item rating scale questionnaire was administered to the participants (never or rarely, sometimes, often and always). We also substituted six months or more duration of each symptom with a period of three months^32^. As a result, having occasional abdominal discomfort or pain in the three months preceding the start of the study, as well as at least two or more of the following conditions, was considered criteria for having IBS disorder; Improvement is occasionally seen with feces, and onset is occasionally related with changes in frequency or type of stool^33^. The following criteria were considered to identify constipation-predominant IBS: (1) occasionally hard or lumpy stools and (2) lack of loose, mushy, or watery stools^34^. Diarrhea-predominant IBS was defined as IBS with occasional watery stools and a lack of hard stools^35^. Mixed IBS was defined as having IBS and both of the following criteria: (1) hard or lumpy stools at least occasionally and (2) loose, mushy or watery stools at least occasionally^36^. These longer-lasting GI symptoms could be an IBS. Other people with IBS were considered unshaped subtypes. IBS severity was assessed by questioning individuals about the severity of their abdominal pain in the last three months. They reported this as mild, moderate, severe, and very severe.

### Assessment of other variables

Information on additional characteristics including age, sex, anthropometric measures (weight, height, and waist circumference (WC), disease history (diabetes and colitis), smoking status, medication use (omeprazole, pantoprazole, ranitidine, cimetidine, famotidine, clidinium, hyoscine, blandola, dimethicone, digestive, pancreatin, antiacid, diphenoxylate, loperamide, nortriptyline, amitriptyline or imipramine, fluoxetine, citalopram, fluvoxamine, and sertraline), and marital status was collected via a self-administered questionnaire. The levels of physical activity were also assessed using the General Practice Physical Activity Questionnaire (GPPAQ)^37^ and the study participants were classified as having ≥ 1 h/week physical activity and having < 1 h/week physical activity. A person's body mass index (BMI) was calculated by dividing their weight (kg) by height in square meter (kg/m^2^). Self-reported anthropometric measurements were found to be reasonable based on a pilot study on 200 subjects. Accordingly, the correlation coefficient for weight, height, WC, and BMI from these self-reported values and the one from measured values was 0.95 (*P* < 0.001), 0.83 (*P* < 0.001), 0.60 (*P* < 0.001), and 0.70 (*P* < 0.001), respectively^38^. Using a pretested questionnaire, we collected information on meal regularity (often or always/never or occasionally), intra-meal fluid intake (≤ 3 glasses/ ≥ 3 glasses), and chewing efficiency (a lot/not a lot) The participants were evaluated in terms of dietary supplement consumption (Fe, Ca, vitamins, and other dietary supplements) (yes/no), exitance of colitis (yes/no), and oral contraceptives drug usage (yes/no). Dental status was also examined and subjects were categorized accordingly as “fully dentate”, “lost 1–5 teeth”, and “lost > 5 teeth”.

### Statistical analysis

In this study, participants were classified based on tertile cut-off points of dietary acid load indices. One-way ANOVA and chi-square tests were used to investigate differences in general characteristics of individuals for continuous and categorical variables. The analysis of covariance (ANCOVA) test was used to compare participants' energy-adjusted dietary intakes across dietary acid load indices tertiles. A binary logistic regression test was performed to estimate the odds of IBS and its subtypes across tertiles of PRAL, DAL, and NEAP scores in a crude and propensity score-adjusted model with potential confounders including age, sex, BMI, marital status, education, physical activity, smoking, diabetes history, medication use, dietary supplements use, meal regularity, chewing sufficiency, intra-meal fluid consumption, frequency of fried food consumption, speed of eating, breakfast skipping, and dental status). ORs and 95% CIs for IBS severity (mild, moderate, severe, and very severe) across tertiles of dietary acid load indices were estimated using multivariable ordinal logistic regression in crude and propensity score-adjusted models. All analyses were carried out with the SPSS software (version 24; SPSS Inc.), and a significance level of 0.05 was considered.

## Results

Table1 presents sociodemographic characteristics, eating habits, and the prevalence of IBS and its subtypes across the tertiles of dietary acid load indices. Our results showed that participants in the highest tertiles of PRAL were less likely to be male (*p* < 0.001), to consume ≥ 3 glasses/day fluid while eating the meals (*p* < 0.001), to have irregular meal pattern (*p* = 0.01), to eat > 3 times/wk fried foods (*p* = 0.01), to have speed of eating < 10 min (*p* = 0.001), and to be frequently user of dietary supplements (*p* = 0.01) compared with those in the lowest tertile. Regarding the DAL score, participants in the highest versus lowest tertile of this score were older (*p* = 0.01) and females (*p* < 0.001), had more BMI (*p* < 0.001), got married (*p* = 0.01), had lower educational status (*p* = 0.02), had often/always irregular meal pattern (*p* = 0.001), consumed < 3 glasses/day fluid while eating the meals (*p* < 0.001), had speed of eating < 10 min (*p* < 0.001), had history of diabetes and colitis disease (*p* = 0.02), used medication (*p* = 0.01) and dietary supplements (*p* < 0.001), and lost > 5 teeth (*p* = 0.02). The participants with greater adherence to the NEAP were younger (*p* = 0.02), had lower BMI (*p* = 0.03), were females (*p* < 0.001), had often/always irregular meal pattern (*p* = 0.01), consumed < 3 glasses /day fluid while eating the meals (*p* = 0.01), had frequent fried food intake < 3 times/week (*p* = 0.003), had lower speed of eating (*p* = 0.001), and had history of supplement use (*p* = 0.01).

**Table 1:** Baseline characteristics of study participants as well as the prevalence of IBS and its subtypes across tertiles of dietary acid load indices^a^.

Variables	Tertiles of energy-adjusted PRAL			*P*-value^b^	Tertiles of energy-adjusted DAL			*P*-value	Tertiles of energy-adjusted NEAP			*P*-value
	T1 (< − 2.60)	T2 (− 2.60–7.46)	T3 (˃ 7.46)		T1 (< 37.97)	T2 (37.97–49.51)	T3 (˃ 49.51)		T1 (< 41.09)	T2 (41.09–51.51)	T3 (˃ 51.51)	
Age (y)	36.7 ± 8.06	35.9 ± 7.57	36.2 ± 7.96	0.06	35.9 ± 7.86	35.9 ± 7.73	36.9 ± 7.84	0.01	36.7 ± 8.05	35.9 ± 7.70	36.1 ± 7.84	0.02
BMI (kg/m^2^)	25.1 ± 3.78	24.7 ± 3.82	24.9 ± 3.86	0.10	24.3 ± 3.72	24.7 ± 3.8	25.8 ± 3.82	< 0.001	25.1 ± 3.82	24.7 ± 3.74	24.9 ± 3.88	0.03
Male (%)	406 (36.2)	416 (37.1)	581 (51.8)	< 0.001	268 (27.4)	381 (35.2)	696 (64.3)	< 0.001	396 (35.4)	445 (39.7)	562 (50.1)	< 0.001
Married (%)	908 (82.0)	880 (80.7)	902 (82.4)	0.32	841 (78.8)	860 (81.1)	903 (85.4)	0.01	904 (82.0)	900 (82.0)	886 (81.1)	0.29
Education (under graduated) (%)	417 (37.2)	435 (38.8)	429 (38.3)	0.72	702 (64.8)	679 (62.7)	639 (59.0)	0.02	682 (60.9)	703 (62.7)	696 (62.1)	0.67
Physical activity (1 h or more than one hour) (%)	138 (12.3)	135 (12.1)	170 (15.2)	0.05	113 (10.4)	127 (11.7)	185 (17.1)	< 0.001	138 (12.3)	152 (13.6)	153 (13.6)	0.58
Smoker or ex-smoker (%)	971 (86.6)	979 (87.4)	948 (84.6)	0.13	944 (87.2)	950 (87.7)	916 (84.6)	0.07	963 (86.0)	985 (87.9)	950 (84.7)	0.10
**Regular meal pattern**												
Never/sometimes	386 (34.9)	442(39.9)	478 (44.4)	< 0.001	380 (35.6)	420 (39.2)	463 (43.6)	0.001	409 (37.0)	431 (39.0)	475 (43.2)	0.01
Often/always	720 (65.1)	667 (60.1)	610 (55.6)		688 (64.4)	651 (60.8)	598 (56.4)		679 (63.0)	675 (61.0)	625 (56.8)	
**Chewing sufficiently**												
A lot	147 (13.2)	152 (13.7)	132 (12.0)	0.19	157 (14.7)	130 (12.1)	133 (12.5)	0.17	153 (13.8)	145 (13.1)	133 (12.1)	0.66
Little or moderate	963 (86.8)	954 (86.3)	967 (88.0)		912 (85.3)	941 (87.9)	930 (87.5)		956 (86.2)	958 (86.9)	970 (87.9)	
Fluid Consumption												
< 3 glasses/meals/day	1052 (96.5)	1079 (98.0)	1046 (95.8)	0.01	1014 (96.9)	1037 (97.5)	1011 (95.8)	< 0.001	1048 (96.6)	1082 (98.0)	1047 (95.7)	0.01
≥ 3 glasses/meals/day	38 (3.5)	22 (2.0)	46 (4.2)		32 (3.1)	27 (2.5)	44 (4.2)		37 (3.4)	22 (2.0)	47 (4.3)	
**Breakfast skipping**												
Yes	79 (7.3)	68 (6.3)	91 (8.4)	0.18	963 (92.9)	974 (93.6)	969 (92.1)	0.43	987 (92.3)	1014 (93.5)	1002 (92.1)	0.38
No	996 (92.7)	1012 (93.7)	995 (91.6)		74 (7.1)	67 (6.4)	83 (7.9)		82 (7.7)	70 (6.5)	86 (7.9)	
**Frequent fried food intake**												
≤ 3 times/wk	937 (86.8)	924 (85.2)	885 (81.6)	0.01	901 (86.4)	868 (83.2)	877 (83.5)	0.003	938 (87.2)	921 (84.6)	887 (81.8)	0.003
> 3 times/wk	143 (13.2)	160 (14.8)	199 (18.4)		142 (5.6)	176 (16.8)	173 (16.5)		137 (2.8)	167 (5.4)	198 (8.2)	
*Speed of eating*												
< 10 min	89 (7.9)	90 (8.0)	134 (12.0)	0.001	92 (8.5)	80 (7.4)	141 (13.0)	< 0.001	91 (8.2)	88 (7.9)	134 (12.0)	0.001
≥ 10 min	1032 (92.1)	1030 (92.0)	987 (88.0)		991 (91.5)	1003 (92.6)	942 (87.0)		1030 (91.8)	1032 (92.1)	987 (88.0)	
Disease history^c^ (%)	29 (2.6)	30 (2.7)	41 (3.7)	0.26	20 (1.8)	33 (3.0)	42 (3.9)	0.02	31 (2.8)	33 (2.9)	36 (3.2)	0.82
Medication use^d^ (%)	80 (7.1)	59 (5.3)	65 (5.8)	0.16	84 (7.8)	50 (4.6)	63 (5.8)	0.01	83 (7.4)	56 (5.0)	65 (5.8)	0.06
Dietary supplement use (%)	360 (32.1)	349 (31.2)	300 (26.8)	0.01	384 (35.5)	349 (32.2)	245 (22.6)	< 0.001	366 (32.7)	346 (30.9)	297 (26.5)	0.01
Dental status^e^ (%)	88 (8.1)	76 (7.0)	90 (8.3)	0.51	79 (7.5)	74 (7.0)	91 (8.7)	0.02	94 (8.6)	72 (6.6)	88 (8.1)	0.40
IBS (%)	232 (20.7)	249 (22.2)	267 (23.8)	0.21	238 (22.0)	248 (22.9)	242 (22.3)	0.87	247 (22.1)	241 (21.5)	260 (23.2)	0.62
IBS-C (%)	78 (7.0)	88 (7.9)	87 (7.8)	0.67	90 (8.3)	81 (7.5)	77 (7.1)	0.56	84 (7.5)	64 (7.5)	85 (7.6)	0.99
IBS-D (%)	57 (5.1)	44 (3.9)	55 (4.9)	0.37	47 (4.3)	48 (4.4)	57 (5.3)	0.53	56 (5.0)	46 (4.1)	54 (4.8)	0.57
IBS-M (%)	31 (2.8)	45 (4.0)	53 (4.7)	0.05	31 (2.9)	47 (4.3)	46 (4.2)	0.13	36 (3.2)	42 (3.7)	51 (4.5)	0.25
IBS-U (%)	66 (5.9)	72 (6.4)	72 (6.4)	0.83	70 (6.5)	72 (6.6)	62 (5.7)	0.64	71 (6.3)	69 (6.2)	70 (6.2)	0.98

PRAL, potential renal acid load; DAL, dietary acid load; NEAP, net endogenous acid production; IBS-C, IBS with constipation; IBS-D, IBS with diarrhea; IBS-M, mixed IBS; IBS-U, unsubtyped IBS.

^a^Data are mean ± SD, unless indicated otherwise.

^b^Obtained from ANOVA or chi-square test, where appropriate.

^c^Chronic disease included: diabetes and colitis.

^d^Medications included omeprazole, pantoprazole, ranitidine, cimetidine, famotidine, clidinium, hyoscine, blandola, dimethicone, digestive, pancreatin, antiacid, diphenoxylate, loperamide, nortriptyline, amitriptyline or imipramine, fluoxetine, citalopram, fluvoxamine, and sertraline.

^e^Dental status > 5 teeth lost.

Daily dietary intakes of study participants across the tertiles of three dietary acid load indices have been summarized in Table 2. In this case, greater PRAL and DAL scores were significantly associated with increased intakes of energy, protein, fat, saturated fatty acid, vitamin D, vitamin E, vitamin B1, vitamin B12, iron, zinc, white meat, red and processed meat, refined grains, and whole grains; as well as decreased intakes of carbohydrate, dietary fiber, vitamin A, vitamin C, vitamin B6, vitamin B9, calcium, fruits, vegetables, nuts/legumes/soy, dairy, tea/coffee, and pickles. Regarding the NEAP score, the results were similar to PRAL and DAL, while a greater score of the NEAP score was not significantly associated with the intake of energy and calcium.

**Table 2:** Dietary intakes of study participants across tertiles of dietary acid load indices^a^.

	Tertiles of energy-adjusted PRAL			*P*-value ^b^	Tertiles of energy-adjusted DAL			*P*-value	Tertiles of energy-adjusted NEAP			*P*-value
	T1 (N = 1005)	T2 (N = 1002)	T3 (N = 980)		T1 (N = 986)	T2 (N = 975)	T3 (N = 939)		T1 (N = 1005)	T2 (N = 998)	T3 (N = 984)	
**Nutrients**												
Energy intake (kcal/d)	2433.3 ± 25.6	2236.0 ± 25.6	2480.4 ± 26.0	< 0.001	2450.3 ± 26.2	2234.4 ± 26.0	2475.3 ± 27.5	< 0.001	2356 .2 ± 25.8	2396.5 ± 25.8	2395.4 ± 26.1	0.46
Carbohydrate (g/d)	312.8 ± 1.43	295.0 ± 1.44	273.9 ± 1.46	< 0.001	313.7 ± 1.47	295.6 ± 1.46	273.4 ± 1.54	< 0.001	307.8 ± 1.48	288.4 ± 1.48	285.7 ± 1.50	< 0.001
Protein g/d)	83.0 ± 0.40	87.3 ± 0.40	94.7 ± 0.41	< 0.001	82.7 ± 0.41	87.2 ± 0.41	95.4 ± 0.43	< 0.001	82.5 ± 0.41	89.1 ± 0.41	93.2 ± 0.41	< 0.001
Fat (g/d)	94.6 ± 0.57	98.2 ± 0.57	103.0 ± 0.58	< 0.001	94.57 ± 0.58	98.4 ± 0.58	103.3 ± 0.61	< 0.001	97.0 ± 0.58	100.7 ± 0.58	98.1 ± 0.58	< 0.001
Fiber (g/d)	25.7 ± 0.16	22.4 ± 0.16	19.7 ± 0.16	< 0.001	25.7 ± 0.17	22.3 ± 0.17	19.8 ± 0.18	< 0.001	25.1 ± 0.17	22.7 ± 0.17	20.0 ± 0.17	< 0.001
SFA (g/d)	22.4 ± 0.18	22.9 ± 0.18	24.4 ± 0.18	< 0.001	22.4 ± 0.18	23.0 ± 0.18	24.5 ± 0.20	< 0.001	23.0 ± 0.18	23.7 ± 0.18	23.1 ± 0.18	0.02
Vitamin A (RAE/d)	596.3 ± 6.12	498.1 ± 6.15	462.9 ± 6.23	< 0.001	591.8 ± 6.34	500.7 ± 6.32	464.3 ± 6.64	< 0.001	595.7 ± 6.05	522.7 ± 6.05	438.5 ± 6.12	< 0.001
Vitamin C (mg/day)	141.0 ± 1.34	92.6 ± 1.34	70.8 ± 1.36	< 0.001	139.5 ± 1.40	93.2 ± 1.39	71.2 ± 1.46	< 0.001	138.2 ± 1.36	97.8 ± 1.36	68.4 ± 1.37	< 0.001
Vitamin D (μg/d)	0.92 ± 0.02	0.98 ± 0.02	1.01 ± 0.02	0.001	0.92 ± 0.02	0.99 ± 0.02	1.01 ± 0.02	0.001	0.95 ± 0.02	1.01 ± 0.02	0.94 ± 0.02	0.01
Vitamin E (mg/d)	20.6 ± 0.20	21.7 ± 0.20	22.1 ± 0.20	< 0.001	20.7 ± 0.19	21.5 ± 0.19	22.3 ± 0.20	< 0.001	21.0 ± 0.18	22.2 ± 0.18	21.1 ± 0.19	< 0.001
Vitamin B1 (mg/d)	1.77 ± 0.02	1.90 ± 0.02	1.88 ± 0.02	0.001	1.78 ± 0.02	1.90 ± 0.02	1.87 ± 0.02	< 0.001	1.69 ± 0.02	1.81 ± 0.02	2.05 ± 0.02	< 0.001
Vitamin B6 (mg/d)	2.09 ± 0.01	1.94 ± 0.01	1.92 ± 0.01	< 0.001	2.08 ± 0.01	1.94 ± 0.01	1.93 ± 0.01	< 0.001	2.09 ± 0.01	2.01 ± 0.01	1.85 ± 0.01	< 0.001
Folate (μg/day)	356.7 ± 2.21	319.1 ± 2.23	285.8 ± 2.26	< 0.001	356.3 ± 2.30	318.7 ± 2.29	287.3 ± 2.41	< 0.001	351.7 ± 2.24	324.8 ± 2.24	285.3 ± 2.26	< 0.001
Vitamin B12 (μg/day)	2.92 ± 0.03	2.90 ± 0.03	3.06 ± 0.03	0.001	2.89 ± 0.03	2.91 ± 0.03	3.10 ± 0.03	< 0.001	3.00 ± 0.03	3.07 ± 0.03	2.82 ± 0.03	< 0.001
Calcium (mg/day)	1031.1 ± 13.2	990.6 ± 13.3	917.7 ± 13.5	< 0.001	1025.8 ± 13.6	994.9 ± 13.5	919.2 ± 14.2	< 0.001	987.1 ± 13.3	960.3 ± 13.3	993.5 ± 13.4	0.17
Fe (mg/day)	16.9 ± 0.11	18.0 ± 0.10	18.0 ± 0.10	< 0.001	16.9 ± 0.10	18.0 ± 0.10	18.0 ± 0.11	< 0.001	16.4 ± 0.10	17.5 ± 0.10	19.0 ± 0.10	< 0.001
Zinc (mg/day)	10.7 ± 0.05	11.0 ± 0.05	11.5 ± 0.05	< 0.001	10.7 ± 0.05	11.01 ± 0.05	11.6 ± 0.05	< 0.001	10.72 ± 0.05	11.3 ± 0.05	11.2 ± 0.05	< 0.001
**Food groups**												
Fruits (g/d)	491.2 ± 5.91	274.9 ± 5.94	183.8 ± 6.02	< 0.001	486.0 ± 6.14	278.8 ± 6.12	184.6 ± 6.44	< 0.001	477.9 ± 6.03	294.5 ± 6.03	177.9 ± 6.10	< 0.001
Vegetables (g/d)	298.5 ± 3.43	225.3 ± 3.44	192.8 ± 3.50	< 0.001	293.2 ± 3.58	228.1 ± 3.56	194.6 ± 3.74	< 0.001	297.1 ± 1.43	233.3 ± 3.41	186.3 ± 3.45	< 0.001
White meat (g/d)	47.4 ± 1.27	57.4 ± 1.27	86.4 ± 1.29	< 0.001	47.0 ± 1.30	57.5 ± 1.29	87.3 ± 1.36	< 0.001	48.2 ± 1.30	62.6 ± 1.30	80.2 ± 1.32	< 0.001
Red and processed meat (g/d)	76.2 ± 1.39	83.4 ± 1.39	93.6 ± 1.41	< 0.001	75.35 ± 1.41	83.1 ± 1.41	95.6 ± 1.48	< 0.001	77.6 ± 1.40	88.50 ± 1.40	87.1 ± 1.41	< 0.001
Nuts, legumes and soy (g/d)	58.7 ± 1.14	58.6 ± 1.14	54.3 ± 1.16	0.01	59.27 ± 1.16	58.0 ± 1.16	54.5 ± 1.22	< 0.001	58.6 ± 1.13	61.02 ± 1.13	52.0 ± 1.15	< 0.001
Refined grains (g/d)	343.2 ± 5.06	414.2 ± 5.08	422.0 ± 5.15	< 0.001	349.7 ± 5.22	411.5 ± 5.20	419.9 ± 5.47	< 0.001	329.0 ± 4.88	386.5 ± 4.88	464.59 ± 4.94	< 0.001
Whole grains (g/d)	34.6 ± 2.37	45.0 ± 2.38	48.3 ± 2.41	< 0.001	35.2 ± 2.45	42.8 ± 2.44	50.7 ± 2.56	< 0.001	39.6 ± 2.38	49.7 ± 2.37	38.3 ± 2.40	0.001
Dairy intake (g/d)	406.9 ± 8.10	341.0 ± 8.13	297.2 ± 8.24	< 0.001	401.2 ± 8.35	344.4 ± 8.31	300.0 ± 8.74	< 0.001	415.2 ± 8.00	360.9 ± 8.00	268.6 ± 8.09	< 0.001
Tea and coffee (g/d)	449.2 ± 8.08	39.1 ± 8.77	300.9 ± 8.93	< 0.001	451.7 ± 9.02	384.8 ± 9.00	305.3 ± 9.45	< 0.001	450.3 ± 8.81	378.6 ± 8.81	316.8 ± 8.91	< 0.001
Pickles (g/d)	11.4 ± 0.55	8.20 ± 0.55	7.27 ± 0.56	< 0.001	10.9 ± 0.55	8.76 ± 0.55	7.07 ± 0.58	< 0.001	11.6 ± 0.55	8.24 ± 0.55	7.05 ± 0.56	< 0.001

PRAL, potential renal acid load; DAL, dietary acid load; NEAP, net endogenous acid production.

^a^Data are means ± standard error (SE), unless indicated.

^b^All values were adjusted for age, sex and energy, except for dietary energy intake, which was only adjusted for age and sex using ANCOVA.

Table 3 presents ORs and 95% CIs of IBS across tertile of dietary acid load indices. In crude mode, being in the highest versus lowest tertile of PRAL was marginally associated with increased odds of IBS in the male population (OR = 1.39; 95% CI = (0.99–1.93); *P* trend = 0.04). After adjusting for propensity score, calculated using a logistic regression model with potential confounders, the results failed to show any significant associations. The highest versus lowest tertile category of other dietary acid load indices (DAL and NEAP) were not significantly associated with odds of IBS in the whole population and even after stratification by gender and BMI in both crude and propensity score-adjusted models.

**Table 3:** Gender- and BMI-stratified ORs and 95% CIs for IBS across tertiles of energy-adjusted dietary acid load indices.

	Tertiles of energy-adjusted PRAL			*P*-trend	Tertiles of energy-adjusted DAL			*P*-trend	Tertiles of energy-adjusted NEAP			*P*-trend
	T1	T2	T3		T1	T2	T3		T1	T2	T3	
	OR	OR (95% CI)	OR (95% CI)		OR	OR (95% CI)	OR (95% CI)		OR	OR (95% CI)	OR (95% CI)	
**Whole population**												
Crude	1	1.09 (0.89–1.34)	1.20 (0.98–1.46)	0.08	1	1.05 (0.86–1.29)	1.22 (0.83–1.25)	0.84	1	0.99 (0.79–1.18)	1.07 (0.88–1.30)	0.52
Adjusted model *	1	1.01 (0.80–1.27)	1.01 (0.81–1.28)	0.92	1	1.01 (0.81–1.26)	0.99 (0.78–1.25)	0.32	1	0.99 (0.79–1.27)	1.01 (0.80–1.26)	0.97
**Male**												
Crude	1	1.01 (0.70–1.45)	1.39 (0.99–1.93)	0.04	1	0.97 (0.64–1.46)	1.19 (0.83–1.72)	0.23	1	1.08 (0.75–1.54)	1.25 (0.89–1.74)	0.18
Adjusted model **	1	0.98 (0.62–1.83)	1.20 (0.80–1.80)	0.34	1	0.90 (0.55–1.46)	1.12 (0.73–1.73)	0.44	1	1.27 (0.82–1.96)	1.13 (0.75–1.71)	0.63
**Female**												
Crude	1	1.14 (0.90– 1.46)	1.19 (0.92–1.54)	0.18	1	1.15 (0.91–1.45)	1.15 (0.87–1.52)	0.24	1	0.94 (0.74–1.21)	1.07 (0.83–1.38)	0.65
Adjusted model **	1	1.04 (0.79–1.36)	1.02 (0.75–1.37)	0.90	1	1.09 (0.84–1.40)	1.09 (0.80–1.49)	0.52	1	0.91 (0.69–1.20)	1.05 (0.79–1.39)	0.80
**BMI < 25 (kg/m**^2^**)**												
Crude	1	1.20 (0.91–1.59)	1.25 (0.94–1.66)	0.13	1	1.18 (0.91–1.54)	1.04 (0.78–1.39)	0.69	1	0.98 (0.74–1.30)	1.08 (0.82–1.43)	0.58
Adjusted model ***	1	1.16 (0.84–1.59)	1.08 (0.78–1.50)	0.64	1	1.13 (0.84–1.51)	1.02 (0.73–1.42)	0.83	1	1.01 (0.74–1.39)	1.05 (0.77–1.44)	0.77
**BMI ≥ 25 (kg/m**^2^**)**												
Crude	1	1.02 (0.75–1.39)	1.23 (0.92–1.65)	0.17	1	0.91 (0.66–1.25)	0.96 (0.71–1.29)	0.83	1	1.03 (0.76–1.39)	1.15 (0.85–1.54)	0.37
Adjusted model ***	1	0.87 (0.62–1.23)	0.99 (0.71–1.39)	0.97	1	0.86 (0.60–1.24)	0.96 (0.68–1.34)	0.85	1	1.02 (0.73–1.44)	1.00 (0.71–1.40)	0.99

PRAL, potential renal acid load; DAL, dietary acid load; NEAP, net endogenous acid production.

*Adjusted for propensity score with confounders including age, gender, physical activity, marital status, education level, smoking, chronic disease, medication use, dietary supplement intake, regular meal pattern, eating rate, chewing sufficiency, breakfast skipping, fluid consumption, fried food intake, dental status, and BMI.

**Adjusted for propensity score with confounders including age, physical activity, marital status, education level, smoking, chronic disease, medication use, dietary supplement intake, regular meal pattern, eating rate, chewing sufficiency, breakfast skipping, fluid consumption, fried food intake, dental status, and BMI.

***Adjusted for propensity score with confounders including age, gender, energy physical activity, marital status, education level, smoking, chronic disease, medication use, dietary supplement intake, regular meal pattern, eating rate, chewing sufficiency, breakfast skipping, fluid consumption, fried food intake, and dental status.

The crude and multivariable ORs and 95% CIs of IBS severity across tertiles of dietary acid load indices are presented in Table 4. Accordingly, the highest compared with the lowest category of PRAL was significantly (OR = 1.54; 95% CI = (1.03–2.32) associated with increased odds of IBS severity in participants with BMI ≥ 25 (kg/m^2^) in the crude model. This score was not significantly associated with odds of IBS severity when the analysis controlled for propensity score. We also could not find any significant associations between greater adherence to the DAL and NEAP scores with odds of IBS severity in the whole population as well as after stratification by gender and BMI in both crude and adjusted model for propensity score.

**Table 4:** Gender- and BMI-stratified ORs and 95% CIs for IBS severity across tertiles of dietary acid load indices.

	Tertiles of energy-adjusted PRAL			Tertiles of energy-adjusted DAL			Tertiles of energy-adjusted NEAP		
	T1	T2	T3	T1	T2	T3	T1	T2	T3
	OR	OR (95% CI)	OR (95% CI)	OR	OR (95% CI)	OR (95% CI)	OR	OR (95% CI)	OR (95% CI)
**Whole population**									
Crude	1	1.24 (0.94–1.62)	1.26 (0.95–1.65)	1	1.05 (0.86–1.29)	1.02 (0.83–1.25)	1	0.99 (0.76–1.30)	1.11 (0.84–1.45)
Adjusted mode*	1	1.08 (0.80–1.47)	1.11 (0.80–1.53)	1	0.99 (0.74–1.34)	1.10 (0.80–1.50)	1	0.95 (0.70–1.29)	0.97 (0.71–1.32)
**Male**									
Crude	1	1.04 (0.62–1.73)	1.22 (0.77–1.94)	1	0.97 (0.64–1.46)	1.19 (0.83–1.72)	1	1.10 (0.67–1.82)	1.11 (0.70–1.77)
Adjusted model**	1	0.68 (0.38–1.21)	0.90 (0.52–1.73)	1	0.71 (0.41–1.24)	0.93 (0.51–1.71)	1	0.89 (0.51–1.54)	0.95 (0.54–1.67)
**Female**									
Crude	1	1.31 (0.95–1.80)	1.32 (0.93–1.86)	1	1.15 (0.91–1.45)	1.15 (0.87–1.52)	1	0.61 (0.69–1.31)	1.15 (0.82–1.62)
Adjusted model**	1	1.77 (0.74–1.55)	0.88 (0.59–1.30)	1	0.92 (0.62–1.38)	0.86 (0.57–1.28)	1	0.94 (0.64–1.36)	0.94 (0.65–1.37)
**BMI < 25 (kg/m**^*2*^**)**									
Crude	1	1.03 (0.70–1.52)	1.01 (0.68–1.49)	1	1.18 (0.91–1.54)	1.04 (0.78–1.39)	1	1.02 (0.69–1.50)	0.99 (0.67–1.46)
Adjusted model***	1	0.98 (0.65–1.48)	1.05 (0.68–1.64)	1	0.89 (0.57–1.41)	1.09 (0.70–1.71)	1	0.92 (0.61–1.39)	0.95 (0.62–1.47)
**BMI ≥ 25 (kg/m**^*2*^*)*									
Crude	1	1.51 (1.01–2.26)	1.54 (1.03–2.32)	1	0.91 (0.66–1.25)	0.96 (0.71–1.29)	1	0.97 (0.65–1.45)	1.22 (0.82–1.81)
Adjusted model***	1	0.95 (0.61–1.48)	0.73 (0.46–1.16)	1	0.94 (0.61–1.45)	0.71 (0.45–1.11)	1	0.98 (0.62–1.55)	0.95 (0.60–1.48)

PRAL, potential renal acid load; DAL, dietary acid load; NEAP, net endogenous acid production.

*Adjusted for propensity score with confounders including age, gender, physical activity, marital status, education level, smoking, chronic disease, medication use, dietary supplement intake, regular meal pattern, eating rate, chewing sufficiency, breakfast skipping, fluid consumption, fried food intake, dental status, and BMI.

**Adjusted for propensity score with confounders including age, physical activity, marital status, education level, smoking, chronic disease, medication use, dietary supplement intake, regular meal pattern, eating rate, chewing sufficiency, breakfast skipping, fluid consumption, fried food intake, dental status, and BMI.

***Adjusted for propensity score with confounders including age, gender, energy physical activity, marital status, education level, smoking, chronic disease, medication use, dietary supplement intake, regular meal pattern, eating rate, chewing sufficiency, breakfast skipping, fluid consumption, fried food intake, and dental status.

Table 5 shows the associations between dietary acid load indices and odds of occurrence of IBS subtypes in crude and different adjusted models. The results indicated a significant positive association between PARL and odds of mixed subtype of IBS in crude (OR = 1.74; 95% CI = (1.11–2.74); *P* trend = 0.02) and propensity score-adjusted model (OR = 1.78; 95% CI = (1.05–3.00); *P* trend = 0.03). Our findings also failed to show any significant associations between DAL and NEAP scores and odds of IBS subtypes in crude and propensity score-adjusted models.

**Table 5:** Crude and multivariable-adjusted ORs and 95% CIs for IBS subtypes across tertiles of energy-adjusted dietary acid load indices.

	Tertiles of energy-adjusted PRAL			*P*-trend	Tertiles of energy-adjusted DAL			*P*-trend	Tertiles of energy-adjusted NEAP			*P*-trend
	T1	T2	T3		T1	T2	T3		T1	T2	T3	
	OR	OR (95% CI)	OR (95% CI)		OR	OR (95% CI)	OR (95% CI)		OR	OR (95% CI)	OR (95% CI)	
**IBS-C**												
Crude model	1	1.14 (0.83–1.56)	1.12 (0.82–1.55)	0.47	1	0.89 (0.65–1.22)	0.84 (0.61–1.16)	0.29	1	1.00 (0.73–1.37)	1.01 (0.74–1.38)	0.94
Adjusted model	1	1.05 (0.72–1.52)	1.14 (0.80–1.63)	0.80	1	0.89 (0.62–1.26)	0.86 (0.60–1.24)	0.41	1	0.90 (0.63–1.30)	0.91 (0.64–1.30)	0.60
**IBS-D**												
Crude model	1	0.76 (0.51–1.14)	0.96 (0.66–1.41)	0.84	1	1.02 (0.68–1.54)	1.22 (0.82–1.82)	0.31	1	0.81 (0.54–1.21)	0.96 (0.65–1.41)	0.84
Adjusted model	1	0.74 (0.48–1.16)	0.83 (0.54–1.30)	0.40	1	0.90 (0.58–1.41)	1.01 (0.65–1.57)	0.97	1	0.94 (0.61–1.47)	0.92 (0.59–1.44)	0.72
**IBS-M**												
Crude model	1	1.47 (0.92–2.34)	1.74 (1.11–2.74)	0.02	1	1.54 (0.97–2.44)	1.50 (0.95–2.39)	0.09	1	1.17 (0.74–1.84)	1.43 (0.93–2.22)	0.10
Adjusted model	1	1.53 (0.90–2.61)	1.78 (1.05–3.00)	0.03	1	0.61 (0.36–1.01)	0.96 (0.61–1.52)	0.06	1	1.38 (0.82–2.32)	1.60 (0.96–2.63)	0.07
**IBS-U**												
Crude model	1	1.10 (0.78–1.55)	1.10 (0.78–1.55)	0.60	1	1.03 (0.73–1.45)	0.88 (0.62–1.25)	0.48	1	0.97 (0.69–1.36)	0.98 (0.70–1.38)	0.93
Adjusted model	1	0.94 (0.63–1.39)	0.91 (0.61–1.36)	0.65	1	0.98 (0.67–1.43)	0.83 (0.55–1.24)	0.37	1	0.97 (0.65–1.43)	0.90 (0.61–1.34)	0.61

PRAL, potential renal acid load; dietary acid load; NEAP, net endogenous acid production; IBS-C, IBS with constipation; IBS-D, IBS with diarrhea; IBS-M, mixed IBS; IBS-U, unsubtyped IBS.

Adjusted model: Adjusted for propensity score with confounders including age, gender, physical activity, marital status, education level, smoking, chronic disease, medication use, dietary supplement intake, regular meal pattern, eating rate, chewing sufficiency, breakfast skipping, fluid consumption, fried food intake, dental status, and BMI.

## Discussion

This large sample size cross-sectional study didn’t show any associations between dietary acid load indices and odds of IBS. Also, these indices were not significantly associated with IBS severity. However, the highest versus lowest tertile of the PRAL score was significantly associated with increased odds of mixed subtype of IBS.

To date, as far as we know no study has looked into the relationship between dietary acid load and IBS; however, its association with other inflammation-related conditions has been investigated previously^19,39–42^. Also, the result of an analysis of the Women’s Healthy Eating and Living (WHEL) study including 3,402 breast cancer survivors showed a positive significant association between dietary acid load (the PRAL score) and serum C-reactive protein levels^23^. Moreover, dietary inflammatory index was positively associated with an increased risk of ulcerative colitis in a case–control study in Iran^43^. Evidence shows elevated levels of proinflammatory cytokines in the plasma of patients with IBS indicating chronic low-grade inflammation which is said to be involved in the pathogenesis of the disease^44,45^. It should be noted that greater intake of white meat, red and processed meat, refined grains, and whole grains (leading to a higher intake of protein and phosphorous); as well as decreased intakes of fruits, vegetables, nuts/legumes/soy, and dairy (leading to lower intakes of magnesium, potassium, and calcium) may justify more acidity and lower alkie levels of the diet among the study participants. On the other side, adherence to this unhealthy dietary pattern led to greater fat intake as a suspected dietary factor related to IBS symptoms^46,47^. Fat intake through increment of gastrocolic reflex^48^ and exaggeration of colonic motor response^49^ is linked to GI symptoms in patients with IBS. Similar to our results, a cross-sectional study of the Singaporean population showed that greater adherence to a Western meal was significantly associated with increased odds of IBS^50^. Another study also indicated lower adherence to a healthy pattern (less consumption of fruits, fish, green-yellow vegetables, and milk) and greater adherence to an unhealthy pattern (high consumption of processed food products) in subjects with IBS compared with the non-IBS group^51^. Besides, in a study of Korean students, those with a greater intake of fatty and salty foods had a higher prevalence of IBS^52^.

In this study, we performed additional analyses to investigate any possible gender and BMI differences related to IBS disease. Our hypothesis was based on the evidence that indicates a higher IBS risk among females than males and in overweight/obese participants than in normal-weight ones^53–55^. However, we didn’t find any gender and BMI differences in the associations between dietary acid load indices and odds of IBS and IBS severity which warranted further research.

Another noticeable finding of this study was the significant (PRAL) and non-significant (DAL and NEAP) associations of dietary acid load indices with odds of mixed type of IBS. However, the two latter scores were not significantly associated they tended to increase the odds. These different results were also shown in the previous research^19,39,40^. A case–control stusy by Ronco et al. showed that the highest versus lowest quartile of the PRAL score but not the NEAP score significantly increased the risk of colorectal cancer^39^. A researcher also found similar results in a cross-sectional study of 3,501 Chinese participants on the association between dietary acid load and odds of hypertension^19^. In contrast to our results, in a case–control study, while the NEAP score was significantly associated with lung cancer risk, the PRAL score showed a non-significant association after adjustment for possible confounders^40^.

In general, it seems the inconsistencies between our findings and findings from other studies resulted from the differences in the study population, kind of dietary intake assessment tools, consideration of confounders in the statistical models, type of study designs, and the culture of a population that can affect the final results. As a result of the limitations of the studies and the lack of studies that investigated the associations between dietary acid load and IBS, more prospective research is required to reject or confirm our findings.

However, the exact mechanism of the effect of dietary acid load on the odds and severity of IBS is unclear, some possible mechanisms can be considered. It has been suggested that dietary acid load is linked to metabolic acidosis, a condition characterized by decreasing blood pH levels^56,57^. Dietary intakes are thought to be related to such conditions as a Western diet rich in animal-based foods is considered a positive acid diet^58^. As a result of metabolic acidosis, tissue damage can occur which is also associated with the initiation of inflammatory responses^23^. Real mechanisms by which inflammatory responses and also acidic conditions triggers visceral hypersensitivity in IBS are not well-known, however, it is suggested to be contributed to the activation of ion channel receptors. One of those is transient receptor potential vanilloid type-1 (TRPV-1) which is activated by acidic conditions (pH < 5.9), inflammatory mediators, heat, and capsaicin^59^. This receptor has been shown to over-express in experimentally intestinal inflamed models and it was found that its expression has a crucial role in the occurrence of visceral hypersensitivity^60^. A previous study demonstrated that IBS patients have more TRPV-1 immunoreactive nerve fibers in their colon^61^. Another mechanism may explain through the involvement of the acid-sensing ion channels (ASIC). These receptors are closed at a pH of 7.4 and are activated when the pH falls below 7.0^62^. However, limited evidence is available in human studies, experimental studies have shown possible roles of these receptors in visceral hypersensitivity in GI disorders with IBS-like features^63–65^. Even though there might be a potential role for acidic conditions concerning the visceral hypersensitivity in IBS, however, further research is needed to show whether a diet with a positive acid balance can lower the pH of the GI tract in such a way that affects ion channel receptors or not.

The current study has some strengths. We investigated the associations between dietary acid load and odds of IBS and severity in a large sample size study. Major confounders were controlled in the models. We included a general population that consisted of the subjects with all grades of IBS who visited or not visited the physician. This study also has some limitations. We measured dietary acid load at a single time point which may not show the long-term associations between dietary acid load and odds of IBS as a chronic disease. Dietary acid load indices were calculated based on a small number of nutrients and thus may not reflect the overall acidity of a diet. We found significant associations in males and who's with a BMI of ˃25 kg/m^2^ therefore the generalizability of the results is limited to these groups. Our dietary acid load indices were not validated against the urine acid status of our population and their validity was only reached in Westerns but not Asians. We used a valid and reliable FFQ, however, this FFQ included 106 food items that may not accurately show the total acidity of the subjects. This might be a reason for the non-significant associations between the indices and odds or severity of IBS in this study. However, using other criteria might leads to higher or lower acidity. This study had a cross-sectional design thus the causality of obtained findings can’t infer and need further prospective trial studies. Although a valid questionnaire, Rome III, was used for the assessment of IBS and its complications the chance of misclassification was unavoidable. We controlled the analyses for several confounders; however, the effects of residual confounders especially unmeasured confounders may not fully eliminate.

According to the study's findings, dietary acid load and IBS may have a probable relationship. In addition, the dietary acid load scores were directly and inversely associated with unhealthy and healthy foods, respectively. However, further research is warranted to confirm the findings.

## Data Availability

The data that support the findings of this study are available from the corresponding author upon reasonable request.
